# Low-Power Memristor for Neuromorphic Computing: From Materials to Applications

**DOI:** 10.1007/s40820-025-01705-4

**Published:** 2025-04-14

**Authors:** Zhipeng Xia, Xiao Sun, Zhenlong Wang, Jialin Meng, Boyan Jin, Tianyu Wang

**Affiliations:** 1 School of Integrated Circuits, Shandong University, Jinan, 250100 People’s Republic of China; 2 Suzhou Research Institute of Shandong University, Suzhou, 215123 People’s Republic of China; 3 National International Innovation Center, Shanghai, 201203 People’s Republic of China; 4 State Key Laboratory of Materials for Integrated Circuits, Shanghai Institute of Microsystem and Information Technology, Chinese Academy of Sciences, 865 Changning Road, Shanghai, 200050 People’s Republic of China

**Keywords:** Memristor, Low power, Multi-value storage, Digital logic gates, Neuromorphic computing

## Abstract

As an emerging memory device, memristor shows great potential in neuromorphic computing applications due to its advantage of low power consumption. This review paper focuses on the application of low-power-based memristors in various aspects. The concept and structure of memristor devices are introduced. The selection of functional materials for low-power memristors is discussed, including ion transport materials, phase change materials, magnetoresistive materials, and ferroelectric materials. Two common types of memristor arrays, 1T1R and 1S1R crossbar arrays are introduced, and physical diagrams of edge computing memristor chips are discussed in detail. Potential applications of low-power memristors in advanced multi-value storage, digital logic gates, and analogue neuromorphic computing are summarized. Furthermore, the future challenges and outlook of neuromorphic computing based on memristor are deeply discussed.
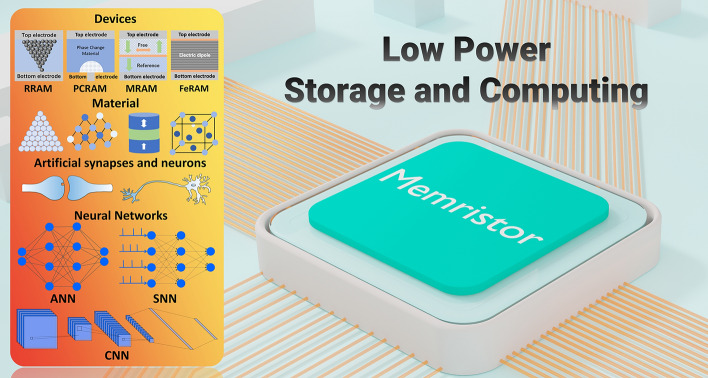

## Highlights

- This review describes various types of low-power memristors, demonstrating their potential for a wide range of applications.
- This review summarizes low-power memristors for multi-level storage, digital logic, and neuromorphic computing, emphasizing their use as artificial synapses and neurons in artificial neural network, convolutional neural network, and spiking neural network, along with 1T1R and 1S1R crossbar array designs.
- Further exploration is essential to overcome limitations and unlock the full potential of low-power memristors for in-memory computing and AI.

## Introduction

Von Neumann architecture is the basic architecture of modern computers, proposed by mathematician John von Neumann in 1945. Its core idea is to store program instructions and data in the same memory block and process the data by reading and executing these instructions through a central processing unit (CPU). This architecture's primary benefit lies in its adaptability and malleability, allowing the computer to undertake various tasks by altering programs stored in its memory [1]. However, von Neumann structure has its inherent flaws, where data storage and computing share the same channel. Such working mode limits processing speed of computer, especially if it uses dynamic random access memory (DRAM) as its primary memory. DRAM access not only requires high energy consumption, but also requires periodic refreshing. During data processing, the processor has to run continuously even while waiting for data, leading to additional energy consumption. As a result, the so-called “energy wall” and “speed wall” are formed.

As internet technology rapidly evolves, the demand for artificial intelligence is experiencing exponential growth. Artificial intelligence has achieved numerous breakthroughs in various domains, including image processing, natural language processing, and big data analysis [2–4]. The amount of data that need to be trained and processed are also increasing daily. To address this problem, complex hardware systems consisting of numerous CPUs and graphics processing units (GPUs) have been developed. As semiconductor technology is approaching its physical limits, Moore's law is also facing failure [5, 6], and researchers must examine the constraints of von Neumann architecture through the lens of computer architecture and software algorithms. In this regard, researchers have proposed various approaches, such as the introduction of multi-level caches [7], the introduction of data streaming [8], and the proposal of in-memory computing. Among emerging technologies, in-memory computing, first conceptualized by W.H. Kautz in 1969 [9], seamlessly integrates computational functions within storage, drastically reducing the delay for data transfer. This integration further leads to reduced power consumption and improved efficiency and is hailed as the next-generation computer architecture poised to transcend the barriers of von Neumann architecture. In recent years, there has been a swift advancement in the development of novel non-volatile memory and in-memory computing technology. With high speed, low power consumption and high-density integration capability, memristor is becoming a research hotspot in in-memory computing fields. Inspired by human brain, memristors with weights updating functions are considered ideal for developing in-memory computing and artificial intelligence [10].

**Fig. 1:**
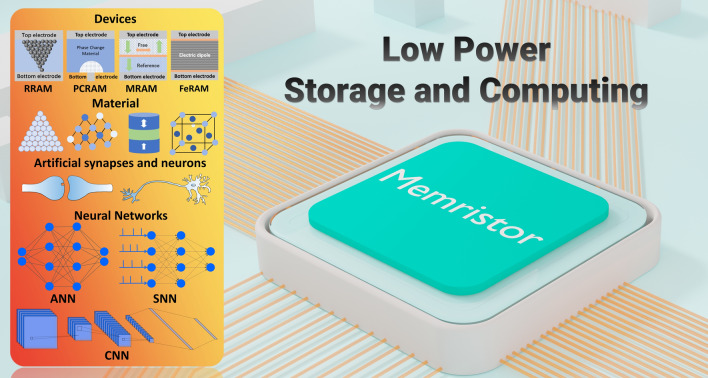
Overview of memristors for low-power storage and computing: including devices, materials, artificial synapses and neurons, and neural networks. From the device level, resistive random access memory (RRAM), phase change random access memory (PCRAM), magnetoresistive random access memory (MRAM) and ferroelectric device are potential low-power neuromorphic computing electronics. From materials system level, ion transport materials, phase change materials, magnetoresistive materials and ferroelectric materials are main functional material layers for low-power memristors. These novel memristors could be used to act as artificial synapses and neurons for low-power neuromorphic computing, including artificial neural network (ANN), spiking neural network (SNN) and convolutional neural network (CNN)

**Fig. 2:**
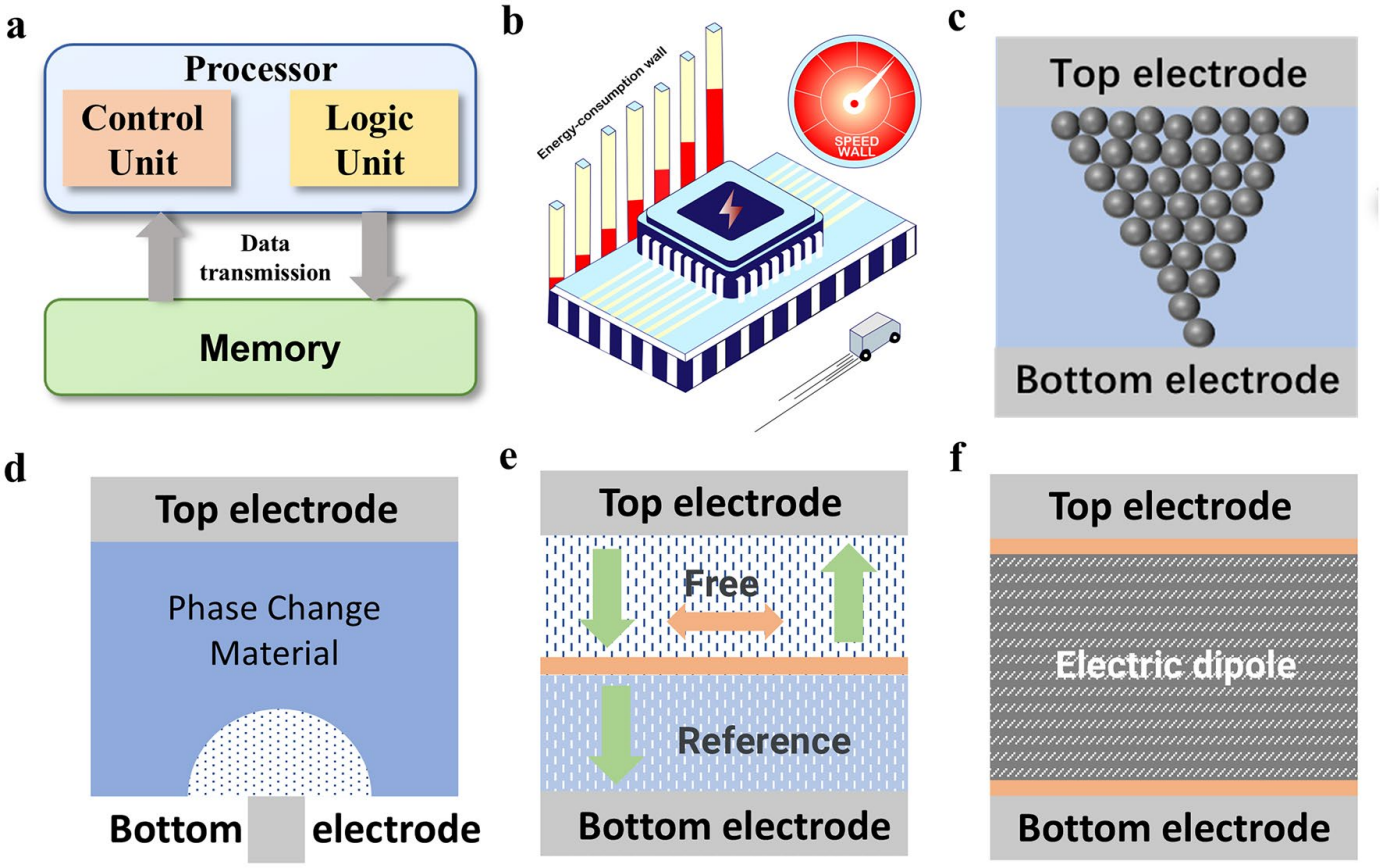
**a** Schematic illustration of the segregation structure. **b** Schematic representation of the “energy wall” and “speed wall” facing the von Neumann structure. **c** Schematic diagram of RRAM device structure. **d** Schematic diagram of PCRAM device structure. **e** Schematic diagram of MRAM device structure. **f** Schematic diagram of ferroelectric device structure

This paper summarizes the research progress of memristors in the field of in-memory computing and artificial intelligence from the perspective of power consumption, covering the aspects of the device structure, mechanism, and key performance parameters of memristors, as well as the introduction of memristor arrays. Then, the low-power functional materials applied in memristors are categorized and discussed. Afterward, the review focuses on discussion of reducing power consumption in several compelling application areas of memristors, especially in multi-bit memories, logic gates, and neuromorphic computing. By summarizing the principles of memristors applied therein, the low-power implementation mechanism is well analyzed. Furthermore, the existing research progress, future challenges and outlook are discussed in detail. Figure 1 shows the overview of this review article. Figure 2a shows the von Neumann architecture diagrams mentioned above. Figure 2b shows a schematic diagram of the “energy wall” and the “speed wall”.

### Memristor

The concept of memristor was first proposed by Professor Chua in 1971, which was the fourth basic passive circuit element after resistance, capacitance, and inductance, filling the gap in the description of the relationship between electric charge and magnetic flux [11]. Its mathematical model is expressed as the ratio of magnetic flux to electric charge,$M = {\text{d}}\varphi /{\text{d}}q$, the resistance is determined by the magnetic flux. It is a nonlinear resistance element with memory characteristics. However, in actual physical systems, direct coupling of magnetic flux and charge is not easy to achieve, and ideal memristors remain more at the theoretical level. Although many devices do not strictly meet the definition of ideal memristors, they exhibit similar characteristics, especially the non-volatile characteristic and adjustability. The realization of generalized memristors is usually based on ion migration, the formation and breaking of conductive filaments (CFs), phase change or magnetic spin effects, etc. RRAM, PCRAM, MRAM and ferroelectric memristor have emerged.

RRAM is one of the typical representatives of memristors. Its resistance state is determined by the distribution of oxygen vacancies or CFs inside the material. The resistance can be changed by voltage pulses and be retained after removing pulses. The structure is usually divided into electrodes and functional layers, presenting a sandwich structure of electrode-functional layer-electrode, as shown in Fig. 2c.

PCRAM uses phase change materials between crystalline and amorphous states to achieve resistance change. The material can be heated to different states under different current pulses, with low resistance in the crystalline state and high resistance in the amorphous state, thereby achieving data writing and storage. The PCRAM device structure is generally mushroom-shaped, with a wider top electrode, a narrower bottom electrode, and a layer of phase change material in the middle. The device structure is shown in Fig. 2d.

MRAM uses the non-volatile magnetic materials and spin electronics for storage. It stores data through a magnetic tunnel junction (MTJ), which consists of two layers of magnetic material and an insulating layer. One magnetic layer is fixed, and the magnetization direction of the other free layer can be changed by current. The resistance state of the MTJ represents the data, with low resistance corresponding to parallel magnetization and high resistance corresponding to antiparallel magnetization. The device structure is shown in Fig. 2e.

Different from early MRAM relying on magnetic field induced switching, spin-transfer torque (STT) technology directly changes the magnetization direction of the free layer through current, reducing power consumption and suitable for high-density storage. Spin-transfer torque random access memory (STT-RAM) is developed based on STT technology. Similarly, there is spin–orbit torque random access memory (SOT-RAM) that uses the spin–orbit torque (SOT) effect.

Ferroelectric memristor uses the polarization characteristics of ferroelectric materials to regulate the resistance state of the device. Ferroelectric materials have reversible polarization direction. When an external electric field is applied, the polarization direction of ferroelectric materials can be flipped, thereby changing the barrier height or interface charge distribution. This change affects the tunneling behavior of the current and the conductivity characteristics and ultimately manifests as different resistance states, as shown in Fig. 2f.

### Functional Materials

**Fig. 3:**
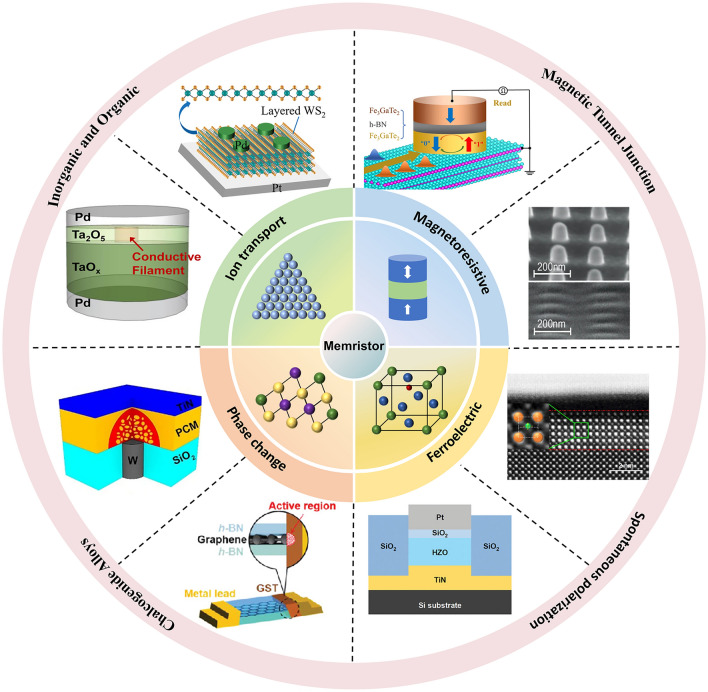
Schematic diagram of memristor classification of different functional materials, including ion transport, phase change, magnetoresistive and ferroelectric. Among them, ion transport materials include organic and inorganic types [33]. Copyright (2014) American Chemical Society [34]. Copyright (2019) Wiley‐VCH, phase change materials are mainly chalcogenide alloys [35]. Copyright (2022) The Authors [36]. Copyright (2020) The Authors, magnetoresistive materials mainly constitute MTJ [37]. Copyright (2023) Science China Press [38]. Copyright (2024) The Authors, and ferroelectric materials mainly have spontaneous polarization characteristics [39]. Copyright (2020) The Authors [40]. Copyright (2024) Wiley‐VCH

According to the common memristor types, memristor functional layer materials can be divided into ion transport materials, phase change materials, magnetoresistive materials and ferroelectric materials, as shown in Fig. 3. Ion transport materials are mainly targeted at RRAM. In recent years, research in this area has mainly focused on inorganic and organic materials, specifically oxides, perovskites, two-dimensional (2D) materials and organic materials. Inorganic oxides have excellent performance and mature preparation technology and are currently widely used, but traditional binary oxides still have problems such as large leakage current and large power consumption. By doping or constructing multi-layer oxide heterojunctions, the formation and dissolution of conductive filaments can be improved for low-power-consumption storage. Perovskites and two-dimensional materials have unique structures, so they have excellent ionic conductivity and low-voltage operation [12, 13, 14, 15]. Organic materials are regarded as strong competitors for the next generation of memory due to their flexibility, adjustability and low-cost potential, especially in flexible devices [16]. Typical research performance reports are summarized in Table 1.

Phase change materials are mainly chalcogenide alloys, with Ge–Sb–Te (GST) as the core. Recent PCRAM devices are also based on GST for heterogeneous doping and proportion alloying. When evaluating the impact of phase change materials on the performance of PCRAM devices, crystallization temperature, thermal conductivity, etc. are key indicators [17]. Khan et al. introduced GeTe/Sb_2_Te_3_ superlattice structure in PCRAM, reducing heat loss and power consumption by 25–30 times [18]. Yang et al. introduced a conductive bridge phase change mechanism into a heterogeneous Ge-Sb-O alloy, which achieved fJ-level energy consumption (43 fJ) [19]. These works provide evidence for low-power-consumption applications of PCRAM.

Magnetoresistive materials with spin polarization characteristics are mainly used for MRAM, where the free layers and fixed layers are made of ferromagnetic materials. As a king of typical ferromagnetic material, CoFeB can form a good interface with the insulating layer and has a low magnetization reversal energy. MgO usually acts as an insulator in the magnetic tunnel junction and can achieve a high tunnel magnetoresistance ratio. Most applications require MTJ to have perpendicular magnetic anisotropy (PMA), that is, the magnetization direction of the material is more likely to be arranged in a direction perpendicular to the plane of the film. PMA is related to the interface effect, lattice structure and stress of the material. The general methods to improve PMA include stacking materials with strong spin–orbit coupling such as ruthenium, cobalt or platinum in the buffer layer, or using an external voltage to regulate the magnetic anisotropy of the magnetic material.

STT-RAM has been partially commercialized, but due to high current requirements and material degradation, researchers introduce SOT-RAM to reduce power consumption and increase write speed through the spin–orbit torque effect. The most studied SOT materials are heavy metal materials and topological insulators with strong spin Hall effect or Rashba effect [20]. Heavy metal materials such as Ta, W and Pt are used for the SOT layer, which have a high spin Hall angle and can efficiently generate spin currents. The surface states of topological insulators (such as Bi_2_Se_3_, Bi_2_Te_3_) have high spin polarization rates and can achieve efficient spin injection at low currents.

**Table 1:** Summary of the characteristics of the four functional materials of RRAM related to device research

Structure	Thickness	Operating voltage	Programming power consumption	Endurance	Year of publication
*Inorganic oxides and heterojunctions*					
ITO/Bi:SnO₂/TiN [21]	20 nm	− 0.5 V/0.4 V	The SET operating power is 16 µW	10⁷	2020
Ag/SiO₂/Ta₂O₅/Pt [22]	6.5 nm	0.14 V to 0.24 V/− 0.06 V to − 0.14 V	N/A	> 1000	2020
Pd/BaTiO_3_:Nd_2_O_3_/La_0.67_Sr_0.33_MnO_3_ (LSMO)/STO [23]	BNO: 34 nm LSMO:12 nm	− 1 V/2 V	0.45 fJ per synaptic event	> 10^10^	2024
*Two-dimensional materials*					
Au/h-BN/Ti [24]	5 nm	− 0.5 V/0.5 V	1.2 pJ/pulse, 30 ns pulse width and 45 µA current	> 6000	2023
Ti /h-BN/Au [25]	~ 2.3 nm	2.75 V	< 2 pJ	600	2024
Pt/WSe_2_/Hf_x_Zr_1−x_O_2_ (HZO)/TiN [26]	WSe_2_: ~ 0.7 nm HZO:10 nm	− 1.2 V / 1.5 V	N/A	> 2000	2025
Au/CuInS2/Cu [27]	N/A	0.6 V	10 nW	1000	2025
*Perovskite materials*					
Ag/CH_3_NH_3_PbI_3_/FTO [28]	350 nm	− 0.2 V/0.2 V	~ 47 fJ μm^−2^	> 10^3^	2020
Ag/BA_2_MA_5_Pb_6_I_19_/Pt [29]	~ 300 nm	− 0.15 V/0.15 V	~ 150 μW, I_cc_ = 1 mA	> 5 × 10^6^	2024
*Organic Materials*					
Al/Cu-doped pMSSQ/Al [30]	~ 80 nm	< 0.9 V	< 0.5 pJ per pulse	500	2017
Ag/PFC-73/ITO [31]	114 nm	0.86 V	N/A	60	2023
ITO/PEDOT:PSS/D:A/PDINN/Ag [32]	The light intensity used (ranging from 0.51 to 194.01 mW cm^−2^)	2023

**Fig. 4:**
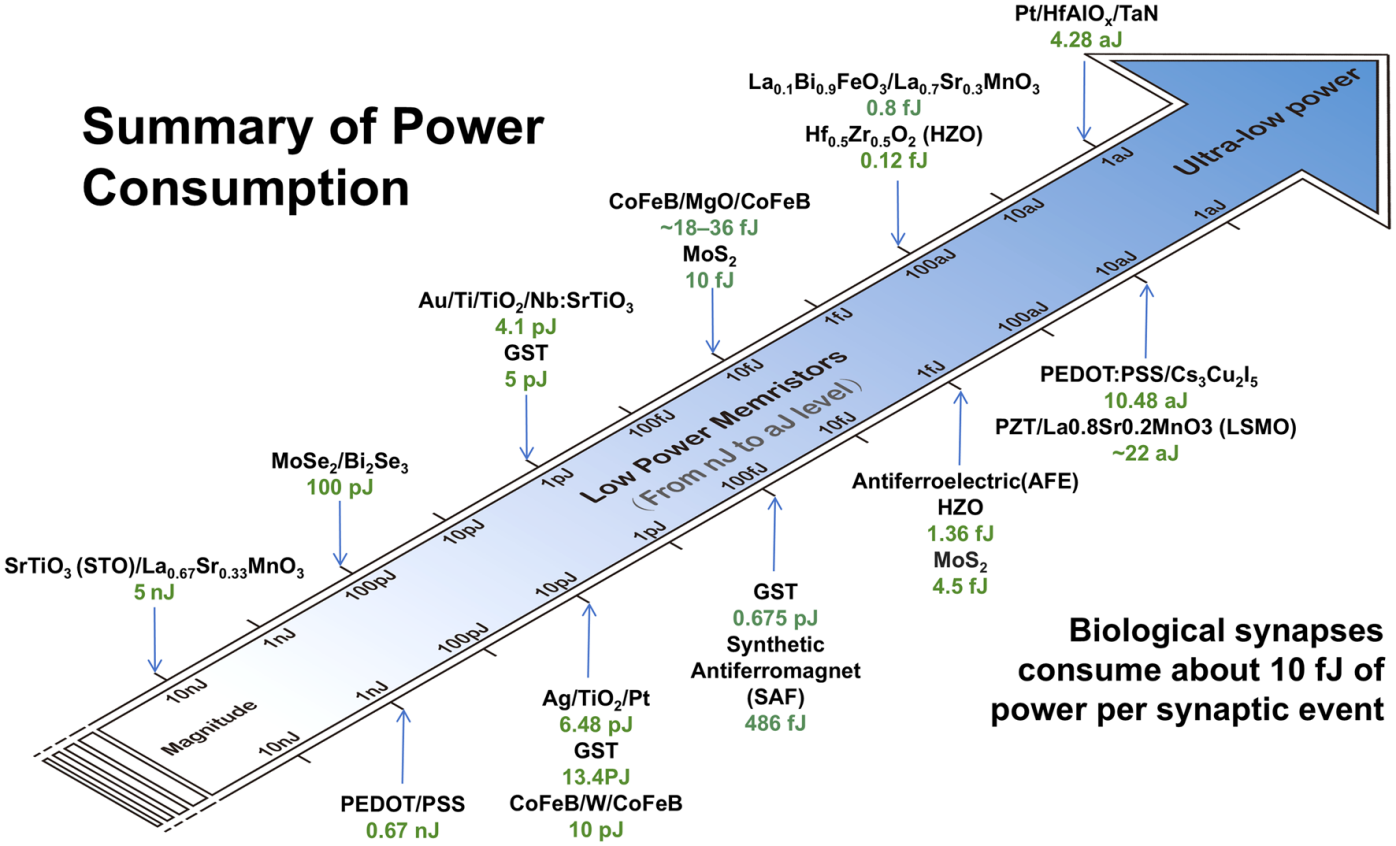
Power consumption of different low-power memristors when performing synaptic plasticity [40–58], where biological synaptic power consumption is ~ 10 fJ. The reported power consumption of novel memristors range from 5 nJ to 4.28 aJ, exhibiting great potential in neuromorphic computing

Ferroelectric materials can achieve reversible polarization reversal under electric field, thereby regulating tunneling current or interface charge distribution and realizing resistance state storage. Classical ferroelectric materials include bismuth titanate (BTO) and barium strontium titanate (BST), which are widely used in ferroelectric tunneling junctions due to their high remanent polarization and low leakage current. Because of excellent complementary metal–oxide–semiconductor (CMOS) compatibility, hafnium oxide-based materials (such as doped HfO_2_) have become a research hotspot in recent years, especially in low-power and high-density memories. Two-dimensional ferroelectrics is a kind of emerging ferroelectric materials, such as In_2_Se_3_ and MoTe_2_, which have ultra-thin thicknesses and are suitable for high-density integration and flexible electronics. Figure 4 summarizes the power consumption of various memristors when completing synaptic operations. RRAM and ferroelectric memristors can reach a lower level than biological levels of 10 fJ. The reported lowest power consumption is 4.28 aJ of HfAlOx-based RRAM, indicating that RRAM exhibits great potential in low-power neuromorphic computing. Therefore, the following content will be expanded on low-power-consumption RRAM.

### Memristor Array

**Fig. 5:**
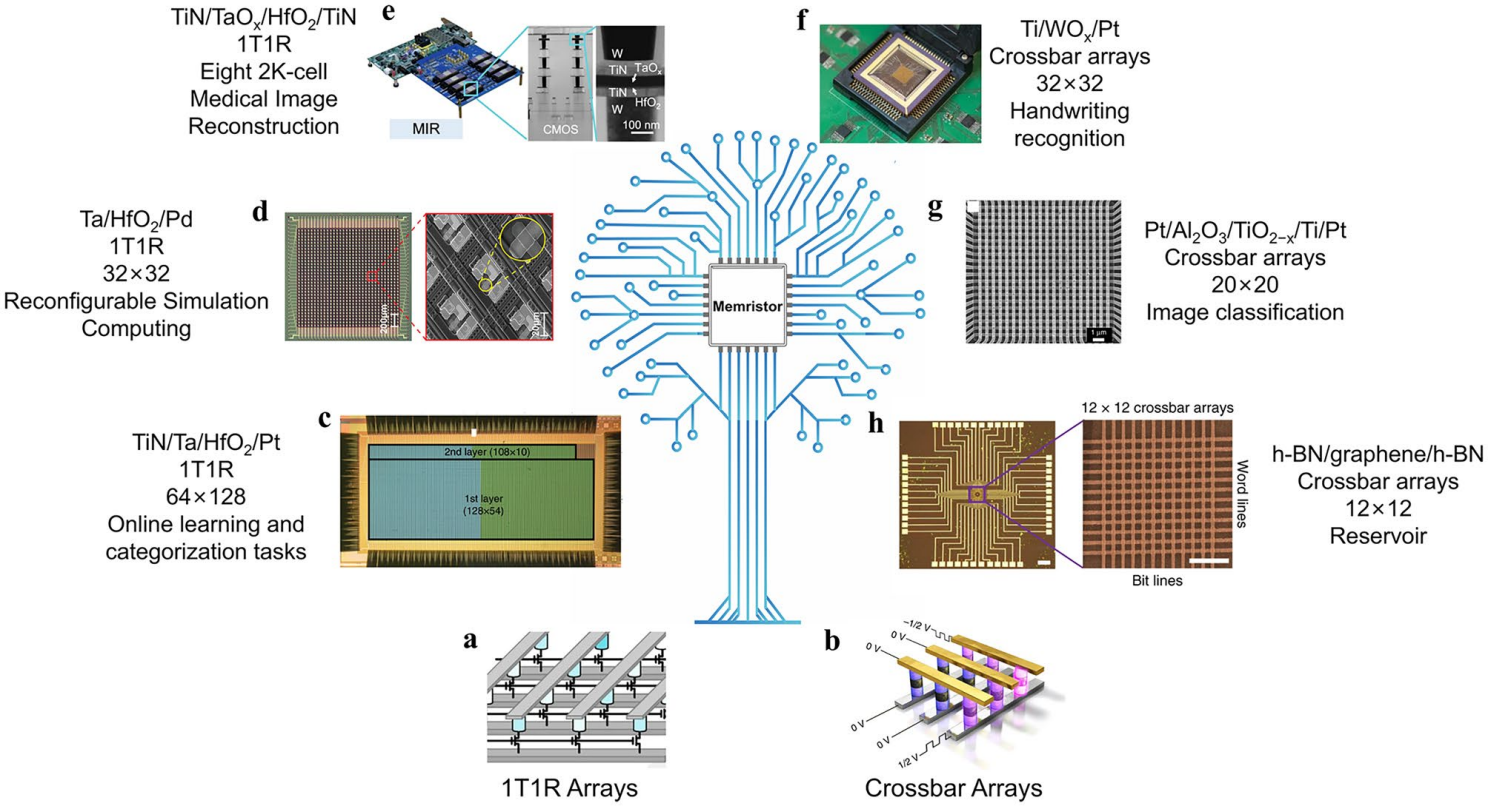
Physical diagram based on 1T1R and crossbar memristor arrays. **a** Schematic diagram of a basic 1T1R array [62]. Copyright (2023) The Authors. **b** Schematic diagram of a basic crossbar array [63]. Copyright (2019) The Authors. **c** 128 × 64 1T1R array for handwritten digit classification [64]. Copyright (2018) The Authors. **d** 32 × 32 1T1R reconfigurable memristor array for analog computing tasks [65]. Copyright (2022) The Authors. **e** 2K memristor chips and an FPGA board, which mainly uses memristor arrays to achieve high-precision medical image reconstruction [62]. Copyright (2023) The Authors. **f** Schematic diagram of 32 × 32 WO_x_ memristor array realize temporal information processing and handwritten digit recognition [66]. Copyright (2017) The Authors. **g** SEM image of a 20 × 20 crossbar array, used for neuromorphic computing with each memristor acting as a synapse [67]. Copyright (2018) The Authors. **h** 12 × 12 crossbar memory array composed of self-selective van der Waals heterostructure memory cells [63]. Copyright (2019) The Authors

Two typical structures of memristor array are the 1 transistor 1 resistor (1T1R) array and the crossbar array. As illustrated in Fig. 5a, 1T1R arrays are active arrays where each memristor is connected in series with a transistor. The word lines connect to the gate electrode of transistor, and the source lines connect to the source of the transistor. The bit lines connect to the top electrode of the memristor, and the bottom electrode connects to the drain of the transistor. The cell area of a 1T1R array is typically 12F^2^ (F is the minimum feature size). As illustrated in Fig. 5b, crossbar arrays are passive arrays with 4F^2^, consisting of perpendicular word lines and bit lines that form a crossbar structure. Memristors are arranged at the cross-points, which is more suitable for integration than a 1T1R and has no quiescent power dissipation. However, crossbar structure is prone to latent path currents. The latent path currents will flow through the other path resistors, thus causing inaccurate readings in the calculations, as well as additional power losses. In contrast, the 1T1R array, with its larger cell area and better isolation of neighboring cells, has no risk of sneak currents, which has higher computational read accuracy. For the crossbar array, a common approach to solving this problem is increasing the *I–V* nonlinearity by connecting a selector in series with one end of each memristor cell. The selector can use either a diode a resistor (1D1R) for unipolar memristors or a two-terminal selector device for bipolar memristors (1S1R). The combined device effectively suppresses the leakage currents caused by the unipolar memristor’s reverse bias or bipolar memristor’s low bias, resulting in much lower currents [59–61]. In recent years, prototype chips based on memristor arrays have been widely developed. Figure 5c–h shows recent studies of memristors arrays, which summarize the structures, the types, the sizes and the realized functions.

## Low-Power Memristor Applications

### Multi-level Storage

A key application of the memristor is non-volatile memory for data storage. The number of states corresponds to discrete resistors that can be read, reflecting the storing ability of memory. Generally, the more storage states there are, the higher the storage density of advanced multi-level memories. In multi-level memories, each resistance state represents a stored value. The dynamic range refers to the ratio of memristor between the maximum conductance and the minimum conductance. Large dynamic range ensures that these states are well separated and differentiated, thereby reducing the possibility of read errors due to noise or drift in the resistance value. In addition, a larger dynamic range also simplifies the peripheral circuit design, which does not need excessively high resolution. Table 2 lists several multi-level storage memristors reported in recent years. In practical applications, as device size decreases or the number of switching cycle increases, material changes and degradation over time also need to be considered. Recent researches focus on improving these aspects in order to achieve higher storage density in memristors.

**Table 2:** Summary of some multi-value memory device materials, state values, dynamic range, operating voltage

Material	State Number	Dynamic Range	Operating Voltages
Hf_x_Zr_1−x_ O_2_ [68]	60	10	± 0.1 V/ ± 2.4 V
Hf_0.5_Zr_0.5_O_2_ (HZO) [69]	8	1500	− 1.6 V/1.4 V
AlO_x_/CeO_x_ [70]	5	22.37	− 1 V/1 V
ZnO [71]	3	6.39	− 0.22 V/0.22 V
HfO_2_/Al_2_O_3_/HfO_2_ [72]	7	10	− 0.3 V/0.8 V
HfO_x_/ZnO [73]	4	330	Less than 3.5 V
Ti_3_C_2_T_x_ MXene [74]	25	10^3^	1.0 V
HfAlO_x_ [75]	5	50	− 2.03 V/2.02 V
NiO [76]	5	10^4^	− 1.23 V/0.79 V
BN [77]	300	10^2^	− 0.79 V/0.81 V
TiO_2_/NiO [78]	4	10^4^	1.0 V
MoS_2_/HfAlO_x_ [79]	6	10^6^	N/A
Ge_2_Sb_2_Te_5_ (GST) [80]	5	~ 13	− 3.5 V/2 V
Y-Sb–Te [81]	3	~ 1000	− 2.5 V/1 V
Ge_2_Sb_2_Te_5_ (GST) [82]	8	200	0.5 V
Pb(Zr,Ti)O_3_ (PZT) [83]	5	29	1.5 V
La:HfO_2_ [84]	8	~ 4	± 1 V/ ± 2.5 V

### Digital Logic Gate

**Fig. 6:**
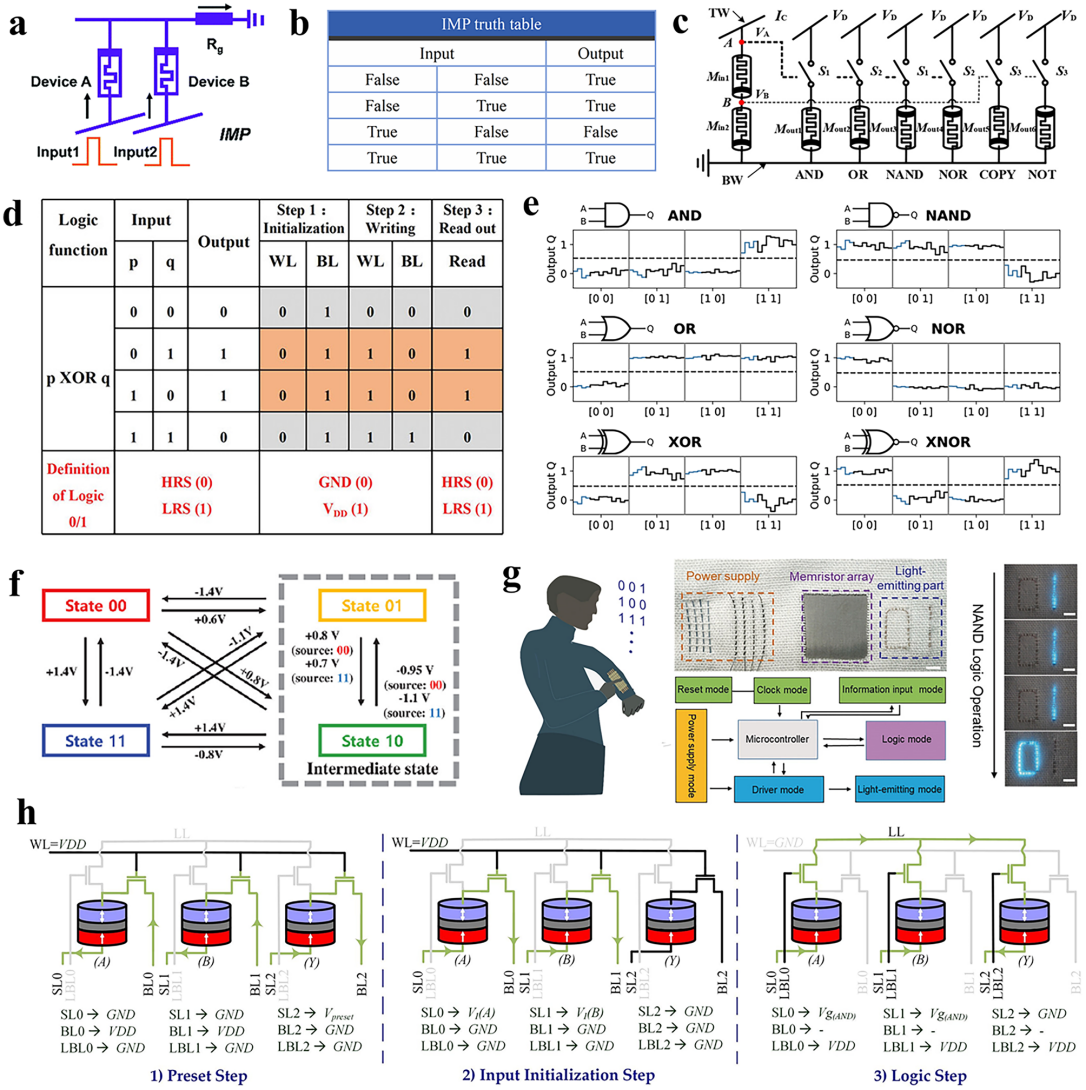
Low-power digital logic gates. **a** Classic IMP gate structure: two memristors in parallel and a series resistor [77]. Copyright (2021) Royal Society of Chemistry. **b** IMP logic gate truth table. “True” is 1, “False” is 0. **c** Schematic diagram of a multi-functional Boolean logic circuit that can simultaneously perform AND, OR, NAND, NOR, COPY, and NOT operations by using a shared set of memristors [88]. Copyright (2020) Institution of Engineering and Technology. **d** The truth table of the XOR logic operation based on memristor of HfSe_2_ [90]. Copyright (2021) The Authors. **e** The output of the linear read-out optimized for various Boolean operations, including AND, NAND, OR, NOR, XOR, and XNOR [91]. Copyright (2022) The Authors. **f** The switching relationships between the four resistance states (00, 01, 10, and 11) in a parallel 2-bit logic-in-memory configuration [92]. Copyright (2023) The Authors. **g** A proof-of-concept all-fabric data-processing system, enabling the real-time output of logic gates [93]. Copyright (2020) Wiley–VCH. **h** The three-step implementation of a stochastic multiplication operation using the 2T-1MTJ IMC method [94]. Copyright (2024) Elsevier

Borghetti et al. [85] first proposed using two memristors in series and parallel to a resistor for implication (IMP) operations. This circuit takes the initial state of the memristor as input and the final state as output, as illustrated in Fig. 6a, where the high-resistance state is '0' and the low-resistance state is '1'. The voltage across the memristor is influenced by 'Input1' and 'Input2' together. By setting Input1 < V_set_ < Input2 and the resistance of memristor B as output, the truth table was obtained as shown in Fig. 6b. Afterward, Kvatinsky et al. [86] developed a memristor-aided logic (MAGIC) gate, integrating two parallel input memristors with a series output memristors. Memristors are installed for input and output separately, circumventing the issue of logic gate's output overwriting the input's value. Huang et al. [87] configured multiple logic functions such as NAND, OR, and XOR by changing the trigger signal without changing the original circuit. Luo et al. [88] implemented two-input or multi-input AND, OR, NAND, and NOR operations, as well as single-input COPY and NOT operations. Other Boolean logic operations are also executable through a mix of IMP operations (Fig. 6c) [89]. In this way, the memristor acts as a logic unit, providing new path for in-memory computing.

The main power consumption issues in the implementation of memristive digital logic are continuous leakage current loss in the off state, dynamic switching energy loss during resistance state transition, and sneak path current in the cross-structure of digital logic. Researchers are mainly looking for solutions in optimizing switching layer materials and developing new architectures. Liu et al. [90] developed a HfSe_x_O_y_/HfSe_2_ device with a low operating voltage of less than 3 V and an operating current of 100 pA. Four-variable sequential logic method was used to achieve various digital logic gates, including XOR, IMP, and NAND, as illustrated in Fig. 6d. Raab et al. [91] studied a single magnetic memristor within a confined triangular geometry, which can perform various digital logic gates including XOR (Fig. 6e). The logic gate has a low current density of about 5 × 10^7 ^A m^−2^, which is four orders of magnitude lower than previous methods and critical for achieving reliable computing with minimal energy consumption. Kho et al. [92] investigated a novel switching phenomenon in HfO_2_ FTJs, precisely controlling the FTJs in four different resistance states (00, 01, 10, and 11), the switching process is shown in Fig. 6f. Xu et al. [93] developed a memristor with a low voltage of 0.3 V and power consumption of 100 pW. The device uses DNA as an active layer binding to Ag nanoparticles. IMP and NAND logic gates were implemented with a series of memristors and pulses (Fig. 6g). Hajisadeghi et al. [94] designed a random memory computing architecture based on STT-RAM, using 2 T-1MTJ units. The detailed steps of random multiplication operations are shown in Fig. 6h. This architecture achieved a 135.7-fold acceleration and a 1.5-fold reduction in energy consumption.

### Artificial Synapse

**Fig. 7:**
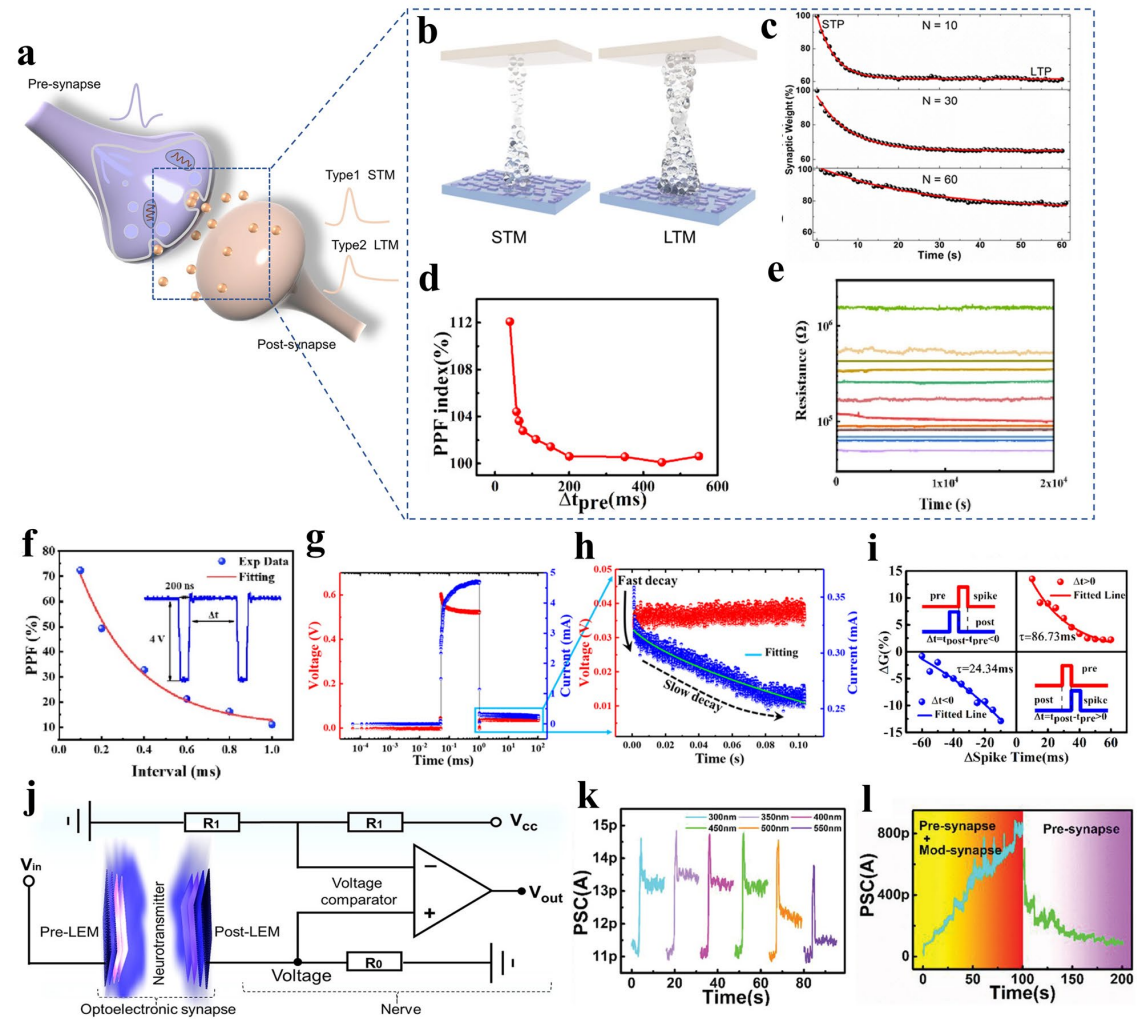
Low-power artificial synapses. **a** Schematic diagram of a biological synapse. **b** The switching mechanism for short-term and long-term memory in an artificial synapse, where the conductance of the memristor changes in response to applied electrical pulses [79]. Copyright (2022) The Authors. **c** The transition from STP to LTP is induced by repetitive pulse stimulation, showing a gradual decay in synaptic weights, consistent with the memory forgetting curve observed in the human brain [98]. Copyright (2017) The Authors. **d** Typical schematic of PPF index variation with time interval [109]. Copyright (2018) American Chemical Society. **e** The 12 multi-level resistive states with long retention times. **f** The PPF index of artificial synapse changes with interval times [99]. Copyright (2023) Elsevier. **g** The EPSC response of artificial device with pulse, indicating the formation and relaxation of CFs. **h** The current decay, which is fitted to a stretched exponential function to model the relaxation process [42]. Copyright (2023) American Chemical Society. **i** Schematic representation of the STDP synaptic learning rule [109]. Copyright (2018) American Chemical Society. **j** The circuit diagram of an optoelectronic artificial efferent nerve system, consisting of a photoelectric synapse with pre- and post-LEMs [104]. Copyright (2021) American Chemical Society. **k** Light control mod-synapse for different excitation wavelengths from 250 to 600 nm. **l** Optoelectronic synergistic control of heterogeneous synaptic potentiation and pure electrical controlled synaptic depression [108]. Copyright (2020) The Authors

Biological synaptic regulation changes the synaptic weights through the presence of specific ions (e.g., Ca^2+^, Na^+^, etc.) inside and outside the cell membrane to achieve the learning and memory functions, as shown in Fig. 7a. Memristors can mimic the plasticity of synapses through adjustable resistance characteristics based on the frequency and strength of the input signal, which play important roles in realizing large-scale neural networks and hardware learning systems [95]. In recent years, researchers have extensively explored various types of memristors and achieved the simulation of a variety of synaptic behaviors, such as short-term plasticity (STP), long-term plasticity (LTP), spike-timing-dependent plasticity (STDP), spike-rate-dependent plasticity (SRDP) and paired-pulse facilitation (PPF) [47, 96, 97].

#### Short-Term/Long-Term Plasticity

The main function of STP in human brain is to process temporary information. LTP is mainly responsible for long-term memory and learning. At the device level, STP and LTP are modeled by applying pulses to the memristor, where the duration of the synaptic weight change defines STP or LTP. This is related to the size of the CFs in the RRAM, as illustrated in Fig. 7b [79], for PCRAM, the degree of crystallization state transition of the phase change material is the decisive factor, which is controlled by the local temperature change caused by Joule heating. In ferroelectric memristors, the influence is the stability and polarization strength of the ferroelectric domain. In STP, when removing the applied voltage, synaptic weights gradually decrease to initial state. LTP refers to the gradual and progressive stabilization of the conductive state under continuous pulses. When removing the applied voltage, the synaptic weight remains stable over time. STP has the potential to be converted into LTP, which can be achieved through repeated stimulation. For example, Wang et al. [98] applied a series of pulses to a memristor (*N* = 10, 30, 60, 90, 120), and the memory retention of the device improved with more pulses, indicating that STP could be converted into LTP through repeated stimulation. The experimental results are illustrated in Fig. 7c.

In biological synapses, PPF mainly reflects the accumulation effect of residual Ca^2^⁺ in the presynaptic neuron. For two adjacent pulse potentials, the first pulse releases neurotransmitters and causes Ca^2+^ influx. The second pulse leads to more Ca^2+^ entry, increasing neurotransmitter release and creating a stronger response in the postsynaptic neuron. PPF is a form of short-term synaptic plasticity, which refers to the phenomenon that when two action potentials (pulses) arrive successively at the presynaptic neuron within a short time interval (10–100 ms), as shown in Fig. 7d. The PPF exponent decreases with the increase of the interval. When the interval exceeds 500 ms, there is no significant difference in the amplitude of the two pulses, indicating that the conductive states have recovered to their initial state. In recent years, many memristors have been able to simulate most functions of biological synapses. For example, Yan et al. [99] designed a ferroelectric memristor with 12 different resistance states (Fig. 7e) for simulating PPF (Fig. 7f). Sahu et al. [42] developed an Ag conductive filaments-based memristor for simulating synaptic plasticity, as shown in Fig. 7g. By applying the voltage of 0.6 V, the CF is formed and the current increased. Subsequently, the current gradually decays due to the reflux of Ag atoms from the CF, as illustrated in Fig. 7h.

#### Spike-Timing-Dependent Plasticity

Hebb proposed a theory in 1949 [100], which states that when two neurons are excited simultaneously, the connection between them will strengthen. STDP builds on this foundation by emphasizing the influence of time sequence, i.e., the regulatory effect of the relative timing of pre- and postsynaptic pulses on synaptic connection strength. The postsynaptic current is enhanced when the stimulation of the presynaptic neuron is earlier than the postsynaptic neuron. On the contrary, the postsynaptic current will be inhibited. At the device level, the time difference between the pre- and postsynaptic neuron pulses can be simulated by controlling the timing sequence of voltage pulses. For example, Fig. 7i shows the actual effect graph, where ΔG expresses the relative conductance before and after the applied pulse. A pre-pulse of − 1.5 V/50 ms and a post-pulse of + 1.5 V/50 ms were applied to the device at t_pre_ and t_pos_, and the time difference between presynaptic neuron and postsynaptic neuron is defined as Δ*t* = *t*_pos_ − *t*_pre_. The results show that the synaptic weights increase for Δ*t* > 0, corresponding to long-term potentiation. On the contrary, the synaptic weight decreases when the current pulse precedes Δ*t* < 0, corresponding to long-term depression in biological synapses.

#### Optoelectronic Synergy and Heterosynapses

In addition to pure electrical control, optical pulses can also modulate the properties of artificial synapses, which offers a richer functionality than a single stimulus [101, 102, 103]. Zhu et al. [104] designed a light-emitting memristor (LEM), which combines the functions of a light receiver, a light transmitter, and an optoelectronic synapse within a single circuit, as illustrated in Fig. 7j. In this system, light signals from the pre-LEM were used as input signals to the post-LEM, realizing dynamically synaptic plasticity. In particular, PCRAM enables multi-level optical state transitions that are highly similar to the plasticity of biological synapses, allowing for rich optical transmission levels by controlling crystallinity [105]. The researchers used optical structure design to reduce optical switching energy consumption. Zhang et al. [106] used a directional coupler structure and phase change material Sb_2_Se_3_ to realize an adjustable optical power divider, which allows an adjustable power distribution ratio from 1 to 97% with zero static power consumption. Nohoji et al. [107] used GST's photonic crystal waveguide for optical neuromorphic synapses, which achieved 81% transmission in the amorphous state and 13% transmission in the crystalline state with low transmission loss.

However, the above studies mainly focused on simple connections in a single device, which is known as homogeneous synapses. In contrast, the synaptic activity of one neuron in neural networks can affect multiple synaptic connections in another neuron, this is classified as a heterogeneous synapse. The role of heterogeneous synapses in neural networks is more complex. Wang et al. [108] developed a multi-type signal modulated artificial synapse that consumes ultra-low energy (< 30 aJ per pulse). By applying electrical and optical signals, excitatory postsynaptic current (EPSC) and biological learning models were mimicked for short-term and long-term memory, as shown in Fig. 7k. Researchers also created four co-modulation modes to enhance synaptic weights modulation range and simulate complex learning algorithms (Fig. 7l).

### Artificial Neuron

**Fig. 8:**
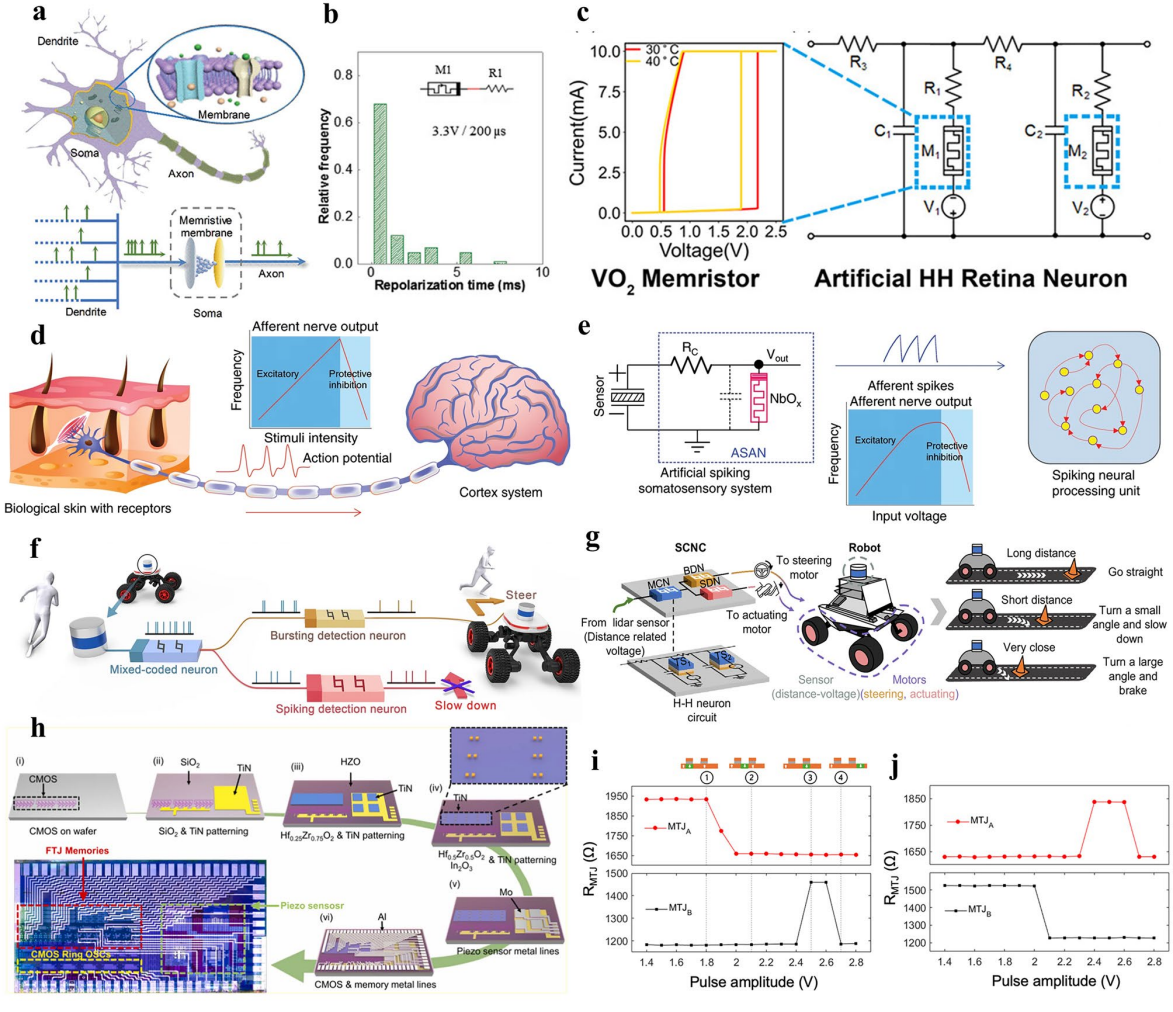
Low-power memristors to construct artificial neurons. **a** Schematic diagram of a biological neuron. **b** The statistical analysis of the self-recovery duration of the Pt/FeO_x_/Ag-based memristors, showing that most recovery processes are completed within 1 ms [125]. Copyright (2018) Wiley‐VCH. **c** VO_2_ memristors constructed for an HH artificial neuron retinal circuit [126]. Copyright (2018) Wiley–VCH. **d** The biological afferent nerve system, where action potentials are generated in the skin and transmitted to the brain, with spiking frequency increasing with stimulus intensity, but decreasing under high stimuli due to protective inhibition. **e** The artificial spiking somatosensory system, which uses a resistor and NbO_x_ memristor to mimic the biological system's frequency response [127]. Copyright (2020) The Authors. **f** Schematic of the neural inspired sensorimotor control neural circuit (SCNC). Two descending memory H–H neurons undergo (Bursting-detection neuron, BDN, Spiking-detection neuron, SDN) decoding distance information, which controls the robot's steering and driving. **g** The robot's obstacle avoidance system, where the input voltage is processed by the SCNC to generate mixed firing patterns in neurons [128]. Copyright (2024) The Authors. **h** The schematic of the fabrication process for a monolithic three-dimensional artificial sensory system, including pressure sensors, FTJ memory, and a silicon-based ring oscillator [129]. Copyright (2024) Elsevier. **i** The relationship between pulse amplitude and resistance states of two devices, where the nucleation of a domain in the domain wall racetrack is followed by a sequence of write, integrate, fire, and reset steps. **j** Similar switching characteristics confirm the directionality of the domain motion during the integration and emission cycles [130]. Copyright (2024) American Chemical Society

A single synapse is not sufficient to perform the complex computations of the brain, and neural network formation is essentially related to the presence of neurons. Figure 8a shows a schematic of biological neuron, inspiring the design of neural circuits by memristors. Memristor-based artificial neuron could reduce the power efficiency significantly compared to previous attempts to build neuron circuits using CMOS [110, 111]. Artificial neurons are constructed to simulate biological characteristics, relying on biophysical neuron models. These models include the Hodgkin–Huxley (H–H) model [112], the leaky integrate-and-fire (LIF) model [113], the FitzHugh–Nagumo model [114], the Morris–Lecar model [115], the Theta neuron model [116], and the Wilson–Cowan model [117], etc. Among these, the H–H model is the most classic type, describing how ion channels affect membrane potential changes. With simplified characteristics, the LIF model focuses on the accumulation of membrane potential and the discharge behavior triggered by the threshold, offering higher computational efficiency [118–124]. Zhang et al. [125] created an artificial neuron on a single memristor that simulates neuronal properties like leakage integration and threshold-triggered excitation. Figure 8b shows that the self-recovery process completes within 1 ms, similar to biological neurons, with an energy consumption of 10 fJ per excitation cycle, comparable to biological neurons. In addition, Xu et al. [126] created an adaptive H–H neuron circuit, as shown in Fig. 8c, which simulates the behavior of biological visual systems under different lighting conditions. By controlling the temperature of the VO_2_ memristors, the circuit mimics the adaptive response of retinal cells and high-frequency firing states. Zhang et al. [127] created a memristor with NbO_x_ layer, utilizing its negative differential resistance to generate neuron-like pulses for spiking neural processing, as shown in Fig. 8d, e.

Yang et al. [128] used NbO_2_ memristors to create a neural circuit model for obstacle avoidance in robots, as shown in Fig. 8f, g. The circuit, based on H–H neurons, processes distance information from light detection and ranging (LiDAR) sensors and enables quick adjustments in the robot's steering and speed. Compared to traditional computing platforms such as GPUs, memristor-based neural circuit reduces latency by more than 50 times and consumes only 5% of the power of traditional platforms. Jung et al. [129] created a monolithic 3D artificial nervous system by integrating piezoelectric sensors, FTJs, and signal processing circuits to simulate sensory neurons for tactile perception (Fig. 8h). The nervous system could detect pressure in the range of 1–50 kPa with a sensitivity of 0.35 mV kPa^−1^, while FTJs can provide frequency-modulated synaptic signals with low power consumption. Cui et al. [130] constructed a spintronic artificial neuron based on a domain wall magnetic tunnel junction, integrating the domain wall inside the magnetic tunnel junction to represent the membrane potential, and reliably performed integration and excitation operations with low power consumption (Fig. 8i, j).

### Artificial Neural Network

**Fig. 9:**
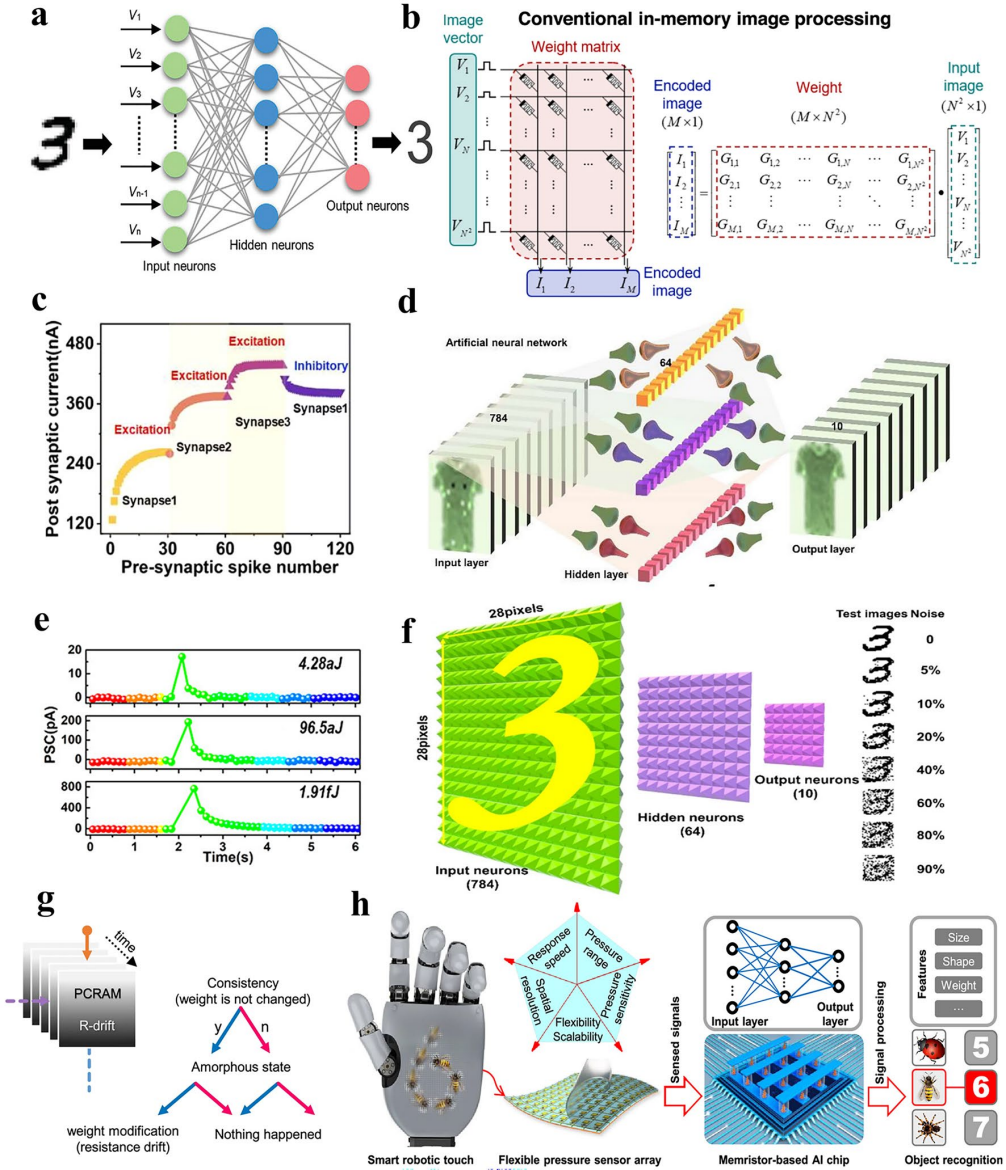
**a** Schematic of a three-layer ANN with input, hidden and output layers [138]. Copyright (2022) The Authors. **b** Schematic illustration of image encoding in a conventional memristor crossbar array based on matrix–vector multiplication, where input voltage is applied to each row [139]. Copyright (2022) The Authors. **c** The spatiotemporal modulation of postsynaptic current in the device using three different presynaptic spikes. **d** Schematic of an ANN constructed for fashion pattern recognition, utilizing the dendritic devices to simulate neural network connections [131]. Copyright (2024) American Chemical Society. **e** Ultra-low-power-consumption characteristics of the memristor, where 4.28 aJ/spike was achieved under voltage pulses of 50 ns. **f** Schematic diagram of the ANN used for MNIST pattern recognition, which consists of an input layer (784 neurons), a hidden layer (64 neurons), and an output layer (10 neurons) [44]. Copyright (2020) American Chemical Society. **g** Schematic showing how resistance drift in PCRAM-based memristors influences weight-change, where the weights increase continuously [132]. Copyright (2021) The Authors. **h** Schematic diagram of an intelligent robotic touch system divided into pressure sensor and memristor computing arrays [137]. Copyright (2022) American Chemical Society

ANN is a computational model formed by simulating biological neural networks. ANNs are composed of input layer, hidden layer and output layer, interconnected to create a complex network structure, as shown in Fig. 9a. The input layer receives external data, which can be mathematical vectors and physical voltage. The hidden layers are located between the input and output layers and are responsible for processing the input data. The computational process is typically a weighted summation, whereby each neuron applies a specific weight value to the received data, sums it, and then through an activation function, converts it into an output signal. During training, the network calculates the error between the output and the actual target, which is called backpropagation. It gradually reduces this error by adjusting the weights of the neuron connections to achieve the target calculation.

In traditional computer systems, neural network weights are stored in RAM, hard disk drives, or solid-state drives as floating-point numbers. The processor reads these weights during calculations and updates them by backpropagation. This process requires many read-and-write operations, which slow down performance and consume energy. In contrast, memristor array stores each weight as a resistance value, allowing for high energy-efficient computing. As shown in Fig. 9b, the input signal is applied to raw of the array as a voltage and is transmitted to column, resulting in a summed output current. The output current is proportional to the product of the input signal and the conductance. In this way, the memristor array performs matrix multiplication directly at the hardware level, replacing large-scale CMOS adders, multipliers, and SRAMs, improving computational efficiency and significantly reducing energy consumption.

Recent studies constructed memristor-based ANNs, where the power consumption is lower than biological levels. For example, Meng et al. [131] developed a dendritic memristor device that simulates synaptic behavior and can communicate through multiple channels. This device can reproduce biological processes, such as Pavlov's conditioning and synaptic cooperation, as shown in Fig. 9c. By adjusting synaptic activities, they created ANNs for pattern recognition, where the device improved recognition accuracy from 91 to 95.2%, as seen in Fig. 9d. This approach in ANNs helps reduce power consumption while enhancing performance. Wang et al. [44] developed a three-dimensional wearable ANN with each spike consuming only 4.28 aJ, far lower than biological energy consumption, as shown in Fig. 9e. The ANN achieves 88.8% recognition accuracy without noise and maintained 80.9% accuracy even with noisy images, as shown in Fig. 9f.

Specifically, PCRAM devices experience spontaneous resistance drift in the amorphous state, where the resistance value gradually increases over time. Resistance drift has been viewed as a reliability issue for PCRAM devices, but Lim et al. [132] proposed a new perspective that makes resistance drift as a spontaneous weight enhancement mechanism. As shown in Fig. 9g, spontaneous resistance drift allows the weights to change over time, reflecting the consistency of the weight state during training. By encoding in this way, the 39 nm PCRAM network automatically controls the sparsity of the weights without additional computational overhead. Based on the results of in-depth research on low-power neural networks, significant breakthroughs have been made in some resource-constrained application scenarios, such as lightweight robots, wearable devices, and the Internet of Things [133–135]. At the same time, combining the memristor neural network with external high-sensitivity sensors creates a lightweight sensing-memory-computing system [136], inspiring new paradigm for the application of memristors. Zhao et al. [137] developed a 64 × 64 flexible tactile sensor array with high-pressure sensitivity and fast response time, integrating it with a computing-in-memory (CIM) chip for recognition tasks. As shown in Fig. 9h, the hardware system achieves accuracy of 98.8% for digits and 97.3% for Chinese characters.

### Convolutional Neural Network

**Fig. 10:**
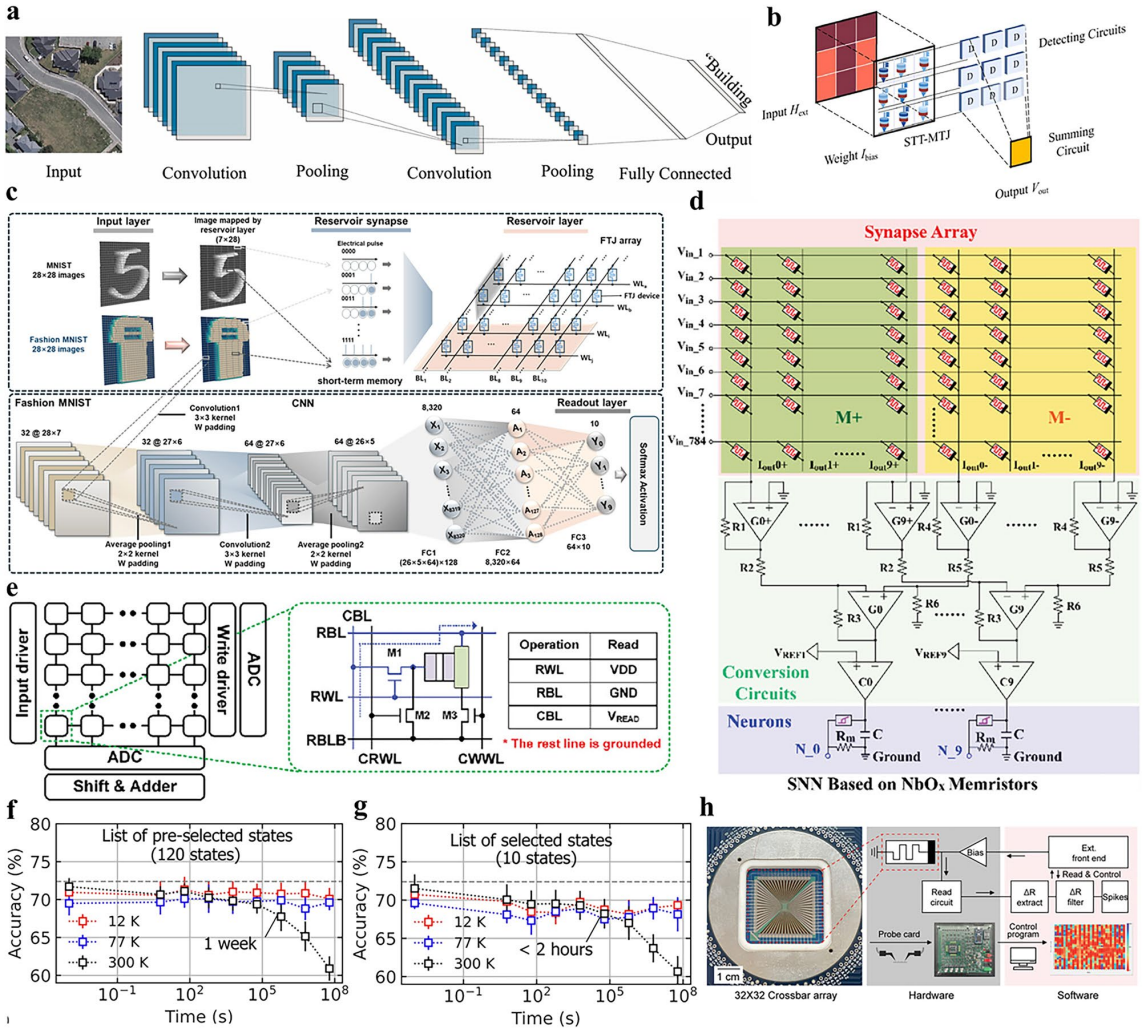
**a** Schematic diagram of the CNN network [144]. Copyright (2024) The Authors. **b** Schematic of in-sensor nonlinear convolutional processing using STT-MTJ arrays [140]. Copyright (2024) Elsevier. **c** CNN model used in a reservoir computing system for pattern recognition, where initial image was mapped through the reservoir layer and training in the read-out layer [142]. Copyright (2025) Elsevier. **d** Schematic of SNN used for MNIST handwritten digit recognition, incorporating synapse arrays and neurons with simulation parameters [145]. Copyright (2023) The Authors. **e** Structure of the device array designed for the SNN using voltage-controlled multi-level MTJs, where the green dotted square represents the MAC operation [146]. Copyright (2024) The Authors. **f** Classification accuracy of the SNN on the MNIST dataset based on PCMs at temperatures of 300 K, 77 K, and 12 K. **g** Performance of the SNN for MNIST classification after 2 years of drift, where drift has a greater impact at room temperature [147]. Copyright (2024) The Authors. **h** Schematic of the SNN hardware testing platform, consisting of a probe card, FPGA-based hardware, and software algorithms [148]. Copyright (2022) Wiley–VCH

CNN is designed to process data with a grid-like structure, such as images. CNNs use convolutional layers to extract features from input data. As shown in Fig. 10a, the data are processed through filters and activation functions, followed by pooling layers to reduce size. The resulting feature maps are flattened and passed through a fully connected layer to generate the final output using functions of Softmax or Sigmoid. Memristor arrays are mainly deployed in convolutional and fully connected layers, which perform multiplication-accumulation operations. As shown in Fig. 10b, the external magnetic field (*H*_ext_) is mapped as the input image, and the bias current (*I*_bias_) is applied to each STT-MTJ device as a weight. The output voltage of STT-MTJ device is added through the summing circuit to form the output of the nonlinear convolution kernel [140]. In the activation layer, normalization layer, and pooling layer, traditional CMOS-based implementations are still used because the operations do not naturally match the analog computing paradigm of memristors. Therefore, the power consumption challenges faced by current memristor-based CNN implementations include not only the losses of the memristor array itself, but also the overhead of peripheral circuits. During the training process of CNN, high-voltage pulse programming operations and high signal-to-noise ratio read operations need to be performed multiple times. In addition, analog memristors are highly dependent on high-precision weighting, which significantly increases the chip area and resistance losses.

In terms of training calculation, Yao et al. [141] integrated eight 1T1R memristor arrays containing 2,048 cells to implement a five-layer CNN with > 96% accuracy on MNIST through a hybrid training methodology, including incorporating initial offline weight parameter establishment, subsequent memristor array mapping, and online fully connected layer retraining of non-idealities. Such architecture demonstrates energy efficiency in two orders of magnitude, which is superior to contemporary GPUs. Lee et al. [142] applied ferroelectric memristor arrays for reservoir computing (RC), the system architecture is shown in Fig. 10c. By adjusting polarization direction, ferroelectric memristors are suitable for multi-dimensional mapping and low-power parallel computing in RC systems. In terms of high precision, Song et al. [143] introduced a novel circuit architecture utilizing multi-stage compensation across memristor subarrays to achieve high-precision computation. The methodology implements sequential error correction through dynamic conductivity matrix programming. The cascaded compensation mechanism mitigates cumulative errors and device variability while maintaining energy efficiency, ultimately achieving numerical computation-grade precision through iterative refinement across the subarray hierarchy.

### Spiking Neural Network

With the extensive development of biological neuroscience, the SNN is gradually demonstrating its outstanding performance, which transmits and processes information through spike signals. In SNN, the activation of a neuron depends not only on the strength of the input signal but also on the timing of the pulse arrival. The introduction of time series enables SNNs to process dynamic, time-sensitive data such as sensory inputs like sound and vision. Another key feature of SNNs is low-power computations for dynamic information. Neurons only emit pulses when necessary and can enter standby mode when there is no input signal. This sparse activity pattern gives it a significant advantage in terms of low power consumption. Describing SNN requires more complex biological dynamic models, this involves the construction of well-designed artificial neuron circuits. However, in the field of algorithm learning, the sequence of pulses introduced by SNN is more difficult to understand than the layer-by-layer calculation method of ANN. Most learning rules of ANN do not apply to SNN, while the most widely adopted rule for SNN is STDP.

Reconfigurable memristor makes it possible to construct SNN with artificial synapses and neurons in the same device array [79]. Based on biological neural models such as H–H or LIF, small-scale SNN based on memristor arrays could be realized. Wang et al. [79] proposed reconfigurable fiber memristor of HfAlO_x_/MoS_2_ functional layer for the first time. With different compliance currents, resistive switching for synaptic behavior and threshold switching for neural behavior were achieved. Han et al. [145] developed a configurable NbO_x_ memristor that functions as either an artificial synapse or neuron based on forming compliance current (FCC). The researchers demonstrated dual functionality through Pavlov's dog experiment and MNIST recognition with accuracy of 91.45%, as shown in Fig. 10d.

Various types of memristor exhibit application potential in SNN. As shown in Fig. 10e, Jeong et al. [146] used multi-layer magnetic states to study spin electronic synapses, achieving energy saving of 28% in SNN operations compared with traditional networks. Palhares et al. [147] studied the performance of GST-based embedded phase change memory (ePCM). At low temperatures, the resistance drift of ePCM is significantly reduced. There is almost no drift at 12 K, ensuring the long-term stability for 2 years. In addition, ePCM in low-temperature environments can reduce power consumption by simplifying the coding scheme while maintaining efficient computing performance. Figure 10f, g shows the impact of ePCM on SNN classification accuracy at different temperatures. Cheong et al. [148] developed a 32 × 32 memristive dot product engine with self-rectifying properties, illustrated in Fig. 10h. The SNN implementation utilized a novel ‘staging system’ that temporarily removes well-trained neuronal connections before re-merging them during inference, which achieved 37% improved energy efficiency with maintained MNIST performance.

### Neural Network Summary

ANNs, CNNs, and SNNs represent different architectural paradigms in neuromorphic computing, each with unique operational characteristics and computational efficiency, as shown in Table 3. ANNs implement fully connected layers of neurons with a feedforward propagation mechanism, utilizing weighted synaptic connections modulated by a backpropagation algorithm, demonstrating robust performance in pattern recognition across different input domains. CNNs utilize spatially localized convolution operations through a hierarchical feature extraction mechanism to implement a shared weight architecture, which is able to efficiently process spatially correlated data through translation-invariant operations and hierarchical feature abstraction. SNNs embody the principles of biomimetic computing through a temporal pulse coding mechanism, achieving energy-efficient asynchronous processing through leaky integration and stimulating neuronal dynamics.

**Table 3:** Summary of technical characteristics of ANN, CNN and SNN

Feature	ANN	CNN	SNN
Architecture	Fully connected layers	Convolutional layers	Spiking neurons with event-driven processing
Energy Efficiency	Moderate (depends on backpropagation)	High (sparse weight matrices and filters)	Great (event-driven and sparse activation)
Training Complexity	Low (backpropagation with gradient descent)	Moderate (backpropagation in convolution layers)	High (based on spike-timing dependent plasticity)
Memristor Role	Weight storage and in-memory computation	Weight storage and in-memory computation	Spike encoding and synaptic weight storage
Low-Power Advantage	Reduction in power consumption for weight updates	In-memory processing for convolutions	Event-based firing reduces unnecessary computation
Use Case	General-purpose tasks, classification	Image processing, object recognition	Event driven tasks, real-time decision

## Conclusion and Perspectives

Overall, memristors represent progress in neuromorphic computing architectures, bringing significant advantages with their inherent physical properties and operational characteristics. First, non-volatile resistance state allows them to store information without additional data transmission power consumption. Second, many memristors achieve stable switching characteristics at feature sizes below 10 nm, with great potential for expansion. In-memory computing eliminates the traditional von Neumann bottleneck and greatly reduces the energy consumption associated with data movement between independent processing and storage units. The adjustable multi-level storage state enables matrix multiplication and weight updates for neuromorphic computing. With excellent CMOS compatibility, memristors facilitate integration into existing semiconductor manufacturing workflows, while supporting new computing paradigms such as logic-in-memory and brain-inspired neuromorphic computing. Recent demonstrations of memristor-based neural networks have achieved remarkable energy efficiencies below 1 fJ per synaptic operation, which is orders of magnitude better than conventional digital implementations and biological computing.

The development of new materials remains key to improving the performance of memristors. Researchers are exploring 2D materials such as graphene and transition metal dichalcogenides, which have unique electrical properties and atomic-level thickness. These materials can achieve more precise resistance modulation and lower power consumption. Research on metal oxides continues, focusing on designing defect states and interface properties to achieve better switching characteristics and reliability. Array structure optimization is to minimize sneak current and improve read/write margins. Advanced selectors can be developed, including volatile switch selectors and engineered tunnel barriers. At the same time, three-dimensional integration strategies are explored to increase storage density while maintaining low power consumption levels.

For storage applications, researchers are developing more complex programming schemes and error correction methods. Research on new switching mechanisms such as phase change and magnetoresistance effects may produce hybrid devices that combine the advantages of different storage mechanisms. For the digital logic computing, future research focuses on optimizing device characteristics for logic operations, developing more efficient programming schemes, and creating new circuit topologies that exploit the unique properties of memristors. In neuromorphic computing, future research will focus on developing ultra-low-power devices and systems, achieving extremely low programming currents to achieve ultra-low-power-consumption pulse generation and transmission. In terms of training schemes, future development schemes need to take into account the non-ideality of the device and optimize the power-performance balance through approximate computing techniques and multi-device architectures.

The development of multi-functional memristor is also advancing, which can perform synaptic and neural functions at the same time, thereby achieving more compact and efficient neuromorphic systems. In response to the challenges of neuromorphic applications, researchers are improving energy efficiency through innovative programming schemes and adaptive precision techniques. Future work will also implement online learning algorithms under low-power operation and explore the use of complementary memristor devices to simplify the weight update process. In addition, the integration of memristor neuromorphic systems with CMOS circuits is also being optimized, especially interface circuits operating at low voltages.
